# A Web-Based Intervention for Health Professionals and Patients to Decrease Cardiovascular Risk Attributable to Physical Inactivity: Development Process

**DOI:** 10.2196/resprot.1804

**Published:** 2012-12-14

**Authors:** Barbara Sassen, Gerjo Kok, Ilse Mesters, Rik Crutzen, Anita Cremers, Luc Vanhees

**Affiliations:** 1 Innovation in Health CareUniversity of Applied SciencesUtrechtNetherlands; 2 Work and Social PsychologyMaastricht UniversityMaastrichtNetherlands; 3 EpidemiologyMaastricht UniversityMaastrichtNetherlands; 4 CahpriMaastricht UniversityMaastrichtNetherlands; 5 Embedded SystemsUniversity of Applied SciencesUtrechtNetherlands; 6 Health and LifestyleUniversity of Applied SciencesUtrechtNetherlands; 7 Rehabilitation SciencesKU LeuvenLeuvenBelgium

**Keywords:** Internet intervention, Intervention Mapping, Health education, Health behaviour change, Health professionals, Cardiovascular risk

## Abstract

**Background:**

Patients with cardiovascular risk factors can reduce their risk of cardiovascular disease by increasing their physical activity and their physical fitness. According to the guidelines for cardiovascular risk management, health professionals should encourage their patients to engage in physical activity.

**Objective:**

In this paper, we provide insight regarding the systematic development of a Web-based intervention for both health professionals and patients with cardiovascular risk factors using the development method Intervention Mapping. The different steps of Intervention Mapping are described to open up the “black box” of Web-based intervention development and to support future Web-based intervention development.

**Methods:**

The development of the Professional and Patient Intention and Behavior Intervention (PIB2 intervention) was initiated with a needs assessment for both health professionals (ie, physiotherapy and nursing) and their patients. We formulated performance and change objectives and, subsequently, theory- and evidence-based intervention methods and strategies were selected that were thought to affect the intention and behavior of health professionals and patients. The rationale of the intervention was based on different behavioral change methods that allowed us to describe the scope and sequence of the intervention and produced the Web-based intervention components. The Web-based intervention consisted of 5 modules, including individualized messages and self-completion forms, and charts and tables.

**Results:**

The systematic and planned development of the PIB2 intervention resulted in an Internet-delivered behavior change intervention. The intervention was not developed as a substitute for face-to-face contact between professionals and patients, but as an application to complement and optimize health services. The focus of the Web-based intervention was to extend professional behavior of health care professionals, as well as to improve the risk-reduction behavior of patients with cardiovascular risk factors.

**Conclusions:**

The Intervention Mapping protocol provided a systematic method for developing the intervention and each intervention design choice was carefully thought-out and justified. Although it was not a rapid or an easy method for developing an intervention, the protocol guided and directed the development process. The application of evidence-based behavior change methods used in our intervention offers insight regarding how an intervention may change intention and health behavior. The Web-based intervention appeared feasible and was implemented. Further research will test the effectiveness of the PIB2 intervention.

**Trial Registration:**

Dutch Trial Register, Trial ID: ECP-92

## Introduction

Developing a Web-based intervention requires a well-thought idea, but also a plan of how to design, implement, and evaluate the intervention. Intervention Mapping provides a framework for building high-quality interventions that are systematically planned, theory- and evidence-based, and take perspectives of end users and intermediaries into consideration [1-6]. Intervention Mapping has been found to be effective for developing interventions [1,2,7-11]. Intervention Mapping consists of 6 planning steps in which each step has a different task and is a prerequisite for the next step. Intervention Mapping places specific emphasis on the transparency of the translation of evidence-based behavior change techniques in intervention components. This is to develop the intervention, explain its rationale, and to facilitate replication [4]. Intervention Mapping is used throughout the process of creating an intervention (from diagnosis of the problem to problem solution) and includes collaborating iteratively with priority groups, stakeholders, and experts in the fields of health education and health promotion.

In designing the intervention for this study, we focused on patients with cardiovascular risk factors. Cardiovascular risk factors increase the risk for cardiovascular disease, Type 2 diabetes, and overall mortality and morbidity [12,13]. Physical activity, particularly intense physical activity, improves cardiovascular fitness and is associated with important cardiovascular health benefits [13,14]. Lifestyle interventions directed at increasing physical activity, thereby enhancing physical fitness, may improve the cardiovascular risk profile of patients. In designing the intervention, we included health professionals, because they can—and should—encourage patients with cardiovascular risk factors to become and stay physically active.

This paper provides insight regarding the systematic development process of the Web-based Professional and Patient Intention and Behavior (PIB2) intervention to open up the “black box” of Web-based intervention development and support future development. The development of the behavioral change intervention should facilitate logic transparency, reproducibility, and diffusion of the intervention. The intervention sought to optimize behavioral coaching by health professionals and to encourage previously physically inactive patients with cardiovascular risk factors to become physically active, following cardiovascular risk management guidelines with a potential for implementation in cardiovascular inpatient and outpatient care [15].

## Methods

**Figure 1:**
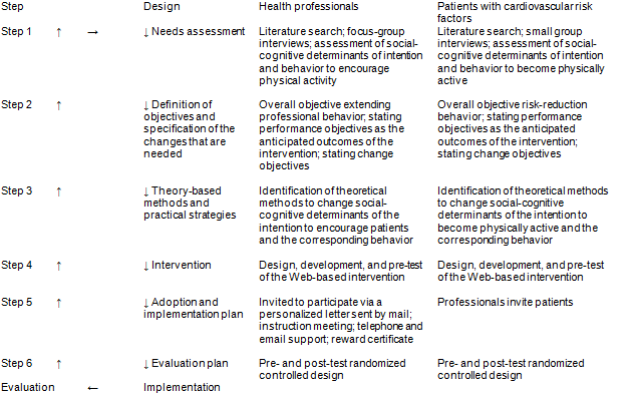
Intervention Mapping steps, design, implementation, and evaluation of the Professional and Patient Intention and Behavior (PIB2) intervention.

The first step of Intervention Mapping is a needs assessment of the study population. The questions explored in the first step included: What is the problem? What are the causes? What behaviors are related to the problem? Are there detectable risk groups? The social-cognitive determinants that could explain intention and behavior were also studied [1,4]. The second step defined the performance objectives with the specification of the change objectives. The performance objectives are the anticipated behavioral outcomes of the intervention. The change objectives describe how important social-cognitive determinants that explain intention and behavior can be changed. In the third step, the performance objectives were linked to the social-cognitive determinants of intention and behavior, as explored in step 1. The theory-based intervention methods to change the determinants of intention and the (health) behavior of interest were selected [1,4]. We selected the theory-based methods, looked at the considerations for using these methods, and described conditions and strategies. Acquiring insight into behavioral change methods to be used in intervention design is essential to evaluate the effectiveness of an intervention because it reveals not only why an intervention changed intention and/or (health) behavior, but also why it may have failed [16,17]. In the fourth step, an intervention was developed based on the integration of these theory-based methods of behavior change and the intervention was pretested. During the fifth step, an adoption and implementation plan for the intervention was created to facilitate sustained implementation. In step 6, the expected results were compared to the actual results to assess the accuracy of the intervention [1,4]. See Figure 1 for an overview of the steps of Intervention Mapping, including the design, implementation, and evaluation of the intervention.

Participants were health professionals and former students of the University of Applied Sciences, Utrecht, the Netherlands. Participants with at least a Bachelor’s degree in nursing or physiotherapy and who had consultations with patients with cardiovascular risk factors were invited to participate. Patients with at least one cardiovascular risk factor (abdominal obesity, high blood pressure, low high-density lipoprotein cholesterol, elevated triglycerides, and elevated blood glucose levels) and low physical activity levels were invited [12,13]. We facilitated sustained implementation of the intervention by rewarding health professionals for extensive use of the Web-based PIB2 intervention with a certificate of 10 European credits. The implementation of the PIB2 intervention was approved by the Ethics Committee at Maastricht University and was registered in the Dutch Trial Register (Trial ID: ECP-92).

## Results

We undertook a separate planning process for health professionals because they perform different behaviors with different social-cognitive determinants related to the intention and behavior in question than their patients with cardiovascular risk factors do [18,19].Within each step of the Intervention Mapping protocol, the application for health professionals is presented followed by the application for patients with cardiovascular risk factors.

### Intervention Mapping Step 1: Needs Assessment (Health Professionals)

In the needs assessment for health professionals, we performed a literature review, held focus group interviews, and studied the social-cognitive determinants that explained intention and behavior. Focus group interviews with health professionals (n = 7) revealed that encouraging patients to become physically active was seen as an integral part of their daily practice and, although perceived as relatively easy to do, patient compliance was often a problem. In previous research, we studied through questionnaire the social-cognitive determinants of intention and behavior to encourage patients with cardiovascular risk factors to become physically active [19-28]. Input for the Intervention Mapping process showed that health professionals’ encouragement of physical activity among cardiovascular patients could be predicted by high levels of intention to encourage physical activity among these patients [19].

### Intervention Mapping Step 1: Needs Assessment (Patients)

In the needs assessment for patients with cardiovascular risk factors, the literature showed various guidelines explaining the recommended levels of physical activity and physical fitness. In a previous study, we investigated physical activity and physical fitness in an adult population and found that the intensity of physical activity was especially important in reducing cardiovascular risk factors [18]. Small group interviews (12 interviews with 3 patients each) revealed that patients with cardiovascular risk factors found being physically active a complex health behavior to incorporate into daily living and it was difficult to maintain. We studied through questionnaire the social-cognitive determinants of patients’ physical activity intentions and the corresponding behavior [20-28]. Input for the Intervention Mapping process showed that behavior was predicted by high levels of intention [18].

### Intervention Mapping Step 2: Define Objectives and Specify Changes (Health Professionals)

We defined the desired behavior of health professionals as encouraging behavior conducive to the health of patients. Health professionals should encourage patients with cardiovascular risk factors to become physically active at increasing levels of intensity as an extension of their professional behavior. This overall objective of extending professional behavior was specified in the health care professionals’ performance objectives. The performance objectives were directed at monitoring their encouraging behavior, formulating explicit plans to encourage their patients, and maintaining and habitually encouraging patients to prevent relapse (Table 1).

**Table 1:** Intervention Mapping step 2 performance objectives for health care professionals and patients with cardiovascular risk factors.

Target group	Performance objectives
Health professionals	Monitor the encouragement of physical activity among patients with cardiovascular risk factors as a prerequisite for a physically active patient
	Formulate explicit plans to encourage physical activity among patients with cardiovascular risk factors
	Identify solutions to diminish the barriers to encouraging physical activity among patients with cardiovascular risk factors
	Formulate explicit plans to cope with difficult situations that occur while encouraging physical activity among patients with cardiovascular risk factors
	Maintain and habitually encourage physical activity among patients with cardiovascular risk factors to prevent relapse
Patients with cardiovascular risk factors	Monitor cardiovascular risks linked to the intensity of physical activity
	Make explicit plans for physical activity
	Identify solutions to diminish barriers to physical activity
	Make explicit plans to cope with difficult situations that occur during physical activity
	Maintain a lifestyle marked by physical activity to prevent relapse

The performance objectives were linked with the social-cognitive determinants of intention and behavior as described in step 1. The link between performance objectives and social-cognitive determinants resulted in a matrix displaying the change objectives for health professionals (Tables 2 and 3). For example, the performance objective that professionals formulate explicit plans to encourage patients was related to the change objectives that health professionals know that planning is important, that they describe their personal benefits for planning, that they feel confident planning the encouragement, and they describe when, where, and how they will encourage patients to engage in physical activity.

**Table 2:** Intervention Mapping step 2 (change objectives) performance objectives for health professionals linked to social-cognitive determinants risk perception, attitudes, and social influence.

Performance objectives health professionals	Risk perception and knowledge	Attitude and outcome expectations	Social influence and skills
Monitors encouragement of physical activity among patients with cardiovascular risk factors as a prerequisite for a physically active patient	Describes the relationship between the professional behavior of encouraging physical activity and health outcomes for patients with cardiovascular risk factors; indicates that cardiovascular risk factors are related to the intensity of physical activity; reports relevant justifications for encouraging patients with cardiovascular risk factors to engage in physical activity	Feels positively about encouraging patients with cardiovascular risk factors to become physically active and the (health) benefits of physical activity; expects that physical activity will decrease cardiovascular risk factors	Describes others as supporting or encouraging patients with cardiovascular risk factors; asks for support; feels confident about handling negative social influence when encouraging patients with cardiovascular risk factors; performs skills necessary to encourage physical activity for cardiovascular patients
Formulates explicit plans to encourage physical activity among patients with cardiovascular risk factors	Knows planning is important for encouraging patients with cardiovascular risk factors to engage in physical activity	Describes personal benefits for planning the encouragement of patients with cardiovascular risk factors to engage in physical activity	Feels confident in planning the encouragement of patients with cardiovascular risk factors to engage in physical activity in regard significant others
Identifies solutions to diminish barriers to encourage physical activity among patients with cardiovascular risk factors	Recognizes negative feelings, thoughts, and actions regarding encouraging patients with cardiovascular risk factors to engage in physical activity that keep him/her from encouraging patients	Describes negative feelings, thoughts, and actions regarding encouraging patients with cardiovascular risk factors to engage in physical activity that keep him/her from encouraging patients	Discusses with colleagues the negative feelings, thoughts, and actions about encouraging patients with cardiovascular risk factors to engage in physical activity that keep him/her from encouraging patients
Formulates explicit plans to cope with difficult situations that occur while encouraging physical activity among patients with cardiovascular risk factors		States that he/she is convinced of the importance of encouraging patients with cardiovascular risk factors to engage in physical activity	
Maintains and habitually encourages physical activity among patients with cardiovascular risk factors to prevent relapse	Indicates that relapse is part of encouraging patients with cardiovascular risk factors to engage physical activity	States benefits of encouraging patients with cardiovascular risk factors to engage in physical activity in the short and long term; states that the best reaction to relapse is to restart	Handles negative social influence (to relapse)

**Table 3:** Intervention Mapping step 2 (change objectives) performance objectives for health care professionals linked to social-cognitive determinants self-efficacy and barriers.

Performance objectives for health professionals	Self-efficacy and skills	Barriers and skills to cope with barriers
Monitors that encouragement of physical activity among patients with cardiovascular risk factors is a prerequisite for a physically active patient	Is confident about encouraging patients with cardiovascular risk factors to become physically active; demonstrates the skills necessary to encourage patients with cardiovascular risk factors to become physically active; demonstrates practical skills necessary to encourage physical activity among patients with cardiovascular risk factors	
Formulates explicit plans to encourage physical activity among patients with cardiovascular risk factors	Describes when, where, and how they will encourage patients with cardiovascular risk factors to engage in physical activity	
Identifies solutions to diminish barriers to encouraging physical activity among patients with cardiovascular risk factors		Handles situations that keep them from encouraging patients with cardiovascular risk factors to engage in physical activity
Formulates explicit plans to cope with difficult situations that occur while encouraging physical activity among patients with cardiovascular risk factors	Demonstrates skills in daily planning for the encouragement of patients with cardiovascular risk factors to engage in physical activity	Incorporates difficult situations in daily planning for the encouragement of patients with cardiovascular risk factors to engage in physical activity
Maintains and habitually encourages physical activity among patients with cardiovascular risk factors to prevent relapse	Is confident in his/her ability to encourage patients with cardiovascular risk factors to engage in physical activity; demonstrates that it is best to restart after relapse; evaluates encouraging behavior	Handles incidental situations that keep him/her from encouraging patients with cardiovascular risk factors to engage in physical activity

### Intervention Mapping Step 2: Define Objectives and Specify Changes (Patients)

In formulating our intervention objectives for patients, we defined the desired behavior as risk-reduction behavior assuming that if an intervention reduces the prevalence of risk factors, it can also reduce the prevalence of disease. Thus, not only should physical activity be encouraged for patients, but also the intensity of their physical activity, resulting in healthy behaviors for patients with cardiovascular risk factors [14]. This overall objective of risk-reduction behavior of the patient was specified in the performance objectives (Table 1). The performance objectives were directed at monitoring cardiovascular risk linked to physical activity, making explicit plans, and maintaining a lifestyle marked by physical activity to prevent relapse. The link between these performance objectives and social-cognitive determinants resulted in a matrix displaying the change objectives for patients with cardiovascular risk factors (Tables 4 and 5). Examples of change objectives related to the performance objective that patients maintain a lifestyle marked by physical activity to prevent relapse was related to the change objectives that patients indicate that relapse is a part of changing lifestyle physical activity, stating the health benefits of physical activity, feeling confident about handling negative social influence, and about being able to perform physical activity, demonstrating an ability to restart and evaluate the behavior, and that they can handle incidental situations that keep them from engaging in physical activity.

**Table 4:** Intervention mapping step 2 (change objectives) performance objectives for patients with cardiovascular risk factors linked to social-cognitive determinants risk perception, attitudes, and social influence.

Performance objectives for patients	Risk perception and knowledge	Attitude and outcome expectations	Social influence and skills
Monitors their cardiovascular risk linked to the intensity of physical activity	Describes relationship between physical activity and health; describes their personal cardiovascular risk; describes that cardiovascular risk factors are related to the intensity of physical activity; indicate relevant reasons for physical activity	Feels positively about the (health) benefits of physical activity; expects that physical activity decreases cardiovascular risk factors	Describes significant others as supporting physical activity; asks for support; feels confident about handling negative social influence; performs social skills necessary for physical activity
Makes explicit plans for physical activity	Knows planning is important for physical activity	Describes personal benefits of planning physical activity	Feels confident in planning physical activity in regard to social circumstances
Patient identifies solutions to diminish barriers to physical activity	Recognizes negative feelings, thoughts, and actions about physical activity, cardiovascular risk factors, the body or self that keep him/her from engaging in physical activity	Describes negative feelings, thoughts, and actions about physical activity, cardiovascular risk factors, the body or self that keep him/her from engaging in physical activity	Discusses negative feelings, thoughts, and actions about physical activity, cardiovascular risk factors, the body or self that keep him/her from engaging in physical activity
Makes explicit plans to cope with difficult situations that occur during physical activity		Expresses being convinced that physical activity is important	
Maintains a lifestyle marked by physical activity to prevent relapse	Indicates that relapse is part of changing lifestyle physical activity	States the health benefits of physical activity in the short and long term; states that the best reaction to relapse is to restart	Feels confident about handling negative social influence (to relapse)

**Table 5:** Intervention mapping step 2 (change objectives) performance objectives for patients with cardiovascular risk factors linked to social-cognitive determinants self-efficacy, and barriers.

Performance objectives for patients	Self-efficacy and skills	Barriers and skills to cope with barriers
Monitors their cardiovascular risk linked to the intensity of physical activity	Is confident about being able to perform physical activity; demonstrates the skills; shows practical skills necessary for physical activity	
Makes explicit plans for physical activity	Describes when, where, and how they will engage in physical activity	
Identifies solutions to diminish barriers to physical activity		Handles situations that keep him/her from engaging in physical activity
Makes explicit plans to cope with difficult situations that occur during physical activity	Demonstrates skills in daily planning for physical activity	Incorporates difficult situations in daily planning for physical activity
Maintains a lifestyle marked by physical activity to prevent relapse	Is confident about being able to perform physical activity; demonstrates that it is best to restart after relapse; evaluates physical activity behavior	Handles incidental situations that keep him/her from engaging in physical activity

### Intervention Mapping Step 3: Theory-Based Methods and Practical Strategies

In step 3, we selected the theory-based methods (Table 6). We applied the theoretical method of risk communication and risk perception to encourage thinking about individual risk and personal vulnerability. This was followed by the generation of attitudinal change and outcome expectations by applying the method of decisional balance to list the pros and cons [29]. We applied the theoretical methods resistance to social pressure and mobilizing others for social support to encourage seeking social support [23]. Providing emotional support or supporting people with information and advice influences the performance of the behavior and is a protective factor for health outcomes [1]. Guided practice to encourage subskill enactment was also facilitated [30]. Interventions that maximize beneficial attitudes, subjective norms, and perceived behavioral control have a substantial impact on intentions [29]. We applied the theoretical method of “action and coping planning.” Action planning can initiate changes in intention. Interventions that provoke alterations in intention have a greater impact on health-related behavior [30-32]. Related to action planning, formulating implementation intentions by preparatory planning when, where, and how health professionals would encourage physical activity among patients with cardiovascular risk factors, as well as determining when, where, and how patients would engage in a physical activity was added to the intervention [33]. When health professionals and patients formulate their own plans (their own implementation intentions), they are more likely to attain the goal and to perform the planned health behavior [34]. This strategy of implementation planning was found to generate a medium-sized effect on health behaviors [35]. Coping planning was initiated to allow health professionals and patients to better cope with putting behavior change into practice [36]. Coping planning was initiated when health professionals and patients identified high-risk situations that might cause them to withdraw from the desired behavior [37]. In addition, health professionals and patients were aided to make their own explicit plans to cope with potential difficult situations that would hinder them from engaging in health behavior or relapse [38].

**Table 6:** Intervention mapping step 3 (theory-based methods) social-cognitive determinants linked to theoretical methods and their conditions.

Determinant	Theory-based method	Considerations for use	Conditions and strategies
Risk perception, knowledge	Risk communication, risk perception	Requires knowledge about the relationship between (health) problem and (risk vs nonencouraging) behavior	Encourage thinking about individual risk and personal vulnerability
Attitude, outcome expectations	Decisional balance	Requires consideration and evaluation of behavior	Encourage listing pro and cons of changing the behavior in the short and long term
Social influence and skills	Resistance to social pressure	Requires social-skill enactment with feedback	Encourage to resist social pressure
	Mobilizing others for social support	Requires a network that can potentially support health behavior	Encourage to seek social support
Perceived behavioral controland skills	Guided practice	Requires subskill enactment with feedback	Encourage subskills practice
	Action planning	Requires specification of when, where, and how to act	Planning behavior change, making a behavior change plan
Barriers and skills to cope	Coping planning	Requires identification of high-risk situations and the practice of coping responses	Put into practice behavior change

In a systematic review and meta-analysis, it was found that the number of behavioral change techniques applied had a positive impact on the total effect size of the intervention [6,39]. An overview of reviews on behavioral change techniques to promote behavior change found that risk communication, use of social support, and self-monitoring of behavior were all shown to be relatively effective [16]. Also, coping and action planning were found to have a sustainable effect on health behavior [6,36]. The intervention provided personalized text messages (with individuals’ names in headings) that were shown to be highly effective in encouraging interaction and support of behavioral change in interventions [6,40].

### Intervention Mapping Step 4: Intervention

A Web-based behavioral change intervention was considered the most appropriate intervention. The intervention aimed to motivate health care professionals to encourage physical activity among their patients and to extend their professional behavior. The intervention also aimed to improve risk-reduction behavior in patients with cardiovascular risk factors. The intervention was not developed as a substitute for face-to-face contact between the health professional and the patient, but as an additional instrument to optimize health services [41]. Research has demonstrated the favorable effects of Internet-delivered interventions, also specific for physical activity interventions [42-46]. Computer-delivered interventions can improve social-cognitive determinants and also enhance health behavior and general health maintenance [42]. An Internet-delivered intervention facilitates the delivery of the intervention to a large group of professionals and patients. Using a website is appealing to the group of interest and has the benefits of making processes visible [47].

**Figure 2:**
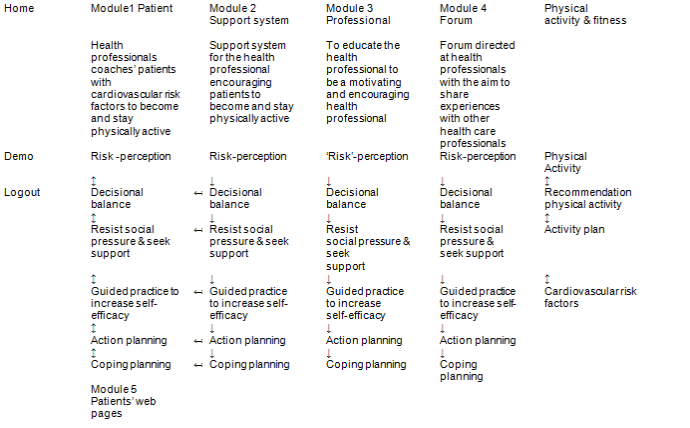
Intervention Mapping step 4 (intervention) flowchart of the intervention.

Several tests and interviews were conducted with experts and members of the target groups (health professionals and patients) to verify the match between intervention components, the performance and change objectives, theory-based methods, conditions for use, and strategies. The PIB2 intervention embeds 5 modules, each comprising a sequential set of screens on the website (Figure 2).

In module 1, the health professionals encouraged the patient to become and stay physically active. Module 1 was designed to invite the patient to participate in the intervention (to use the intervention based on the mutual exchange of information between the health professionals and the patient) paralleling the performance and change objectives, the methods, conditions, and strategies (Figure 2). It started with the assessment of cardiovascular risk factors according to the guidelines for cardiovascular risk management [15]. Based on this assessment, the patient’s cardiovascular risk factors were displayed in a pie chart and changes in the number of risk factors or risk factor levels were registered and also displayed. During the assessment step, the levels of physical activity (intensity and duration) were assessed with the short questionnaire to assess health-enhancing physical activity (SQUASH). Physical activity and the intensity (total intensity and high, medium, and low intensity) of physical activity was displayed in a bar chart; changes were registered and also displayed [48]. The social-cognitive determinants of the patient, and the intention and behavior toward physical activity, were assessed through a questionnaire and displayed in the health spider chart of the patient; changes were registered and later shown [18].

After the assessment, the health professionals began coaching the patient with cardiovascular risk factors through a process of behavior changes to become and to stay physically active. In the process of behavior change, the 7 theory-based methods, conditions, and strategies were put into practice through 7 different website screens. The process of behavior change started with risk perception by encouraging the patient to think about individual cardiovascular risk and personal vulnerability, and the relationship between physical activity and cardiovascular risk. This was followed by listing the pros and cons of changing (or not changing) their behavior in the short and long term, and encouraging the patient to describe what his/her personal pros and cons are to becoming (or not becoming) physically active in the short and long term. After this, the patient was encouraged to resist social pressure and to seek social support, and to practice the necessary subskills. The process of behavior change ended with planning the behavior change by making a behavior change plan and putting the behavior change into practice. The patient was encouraged to specify when, where, and how to become physically active in a plan. When the patient started to put the physical active lifestyle into practice, high-risk situations should be detected and the practice of coping responses was encouraged.

A feedback system measured the progress of the patient’s cardiovascular risk factors, physical activity levels, and the process of behavior change. The patient’s profile was displayed while the health professional was working with the patient. The health professional provided the patient with physical activity recommendations based on research [14,49]. The physical activity recommendations were also shown in the patient’s profile and could be changed by the health professional when needed.

Module 2 was the health professionals’ support system. The support system was parallel to the website screens of module 1 (Figure 2). Module 2 contained background information about how to coach the patient with cardiovascular risk factors through the processes of behavior change. This module consisted of explanations of the methods, conditions, and strategies for the health professionals, scripts on how to start conversations, and so on. The health professionals could read background information for each method and select parts of this information. Once selected, the website displayed the selected background information for all subsequent consultations with this and other patients by clicking the “show suggestions” button in module 1.

Module 3 facilitated the professional to become a motivating and encouraging health professional (Figure 2). It contained self-complete forms and was designed to educate the health professional. The process of behavior change for health professionals started with “risk” communication, thinking about encouraging patients, and thinking about compliant patients. This was followed by listing the pros and cons of encouraging (or not encouraging) patients in the short and long term. After this, the health professional was encouraged to seek social support and look at the subskills needed to be an encouraging health professional. The process of behavior change ended with planning the behavior change, making a behavior change plan, and putting the behavior change into practice. The professional was encouraged to specify in a plan when, where, and how to encourage patients. When the professional started to put his encouraging behavior into practice, the identification of high-risk situations and the practice of coping responses was encouraged. In module 3, a feedback system was incorporated to enable the professional to evaluate their progress. At the initiation of the behavior change process, the health professionals filled in a questionnaire on social-cognitive determinants, intention, and behavior to encourage patients. Progress was registered and made visible in the health professionals’ coaching spider chart [19].

Module 4 consisted of a forum directed at health professionals. Health professionals could use the forum to share experiences with other health professionals who also used the PIB2 intervention and find solutions for specific problems pertaining to the coaching of patients (Figure 2). A researcher responsible for the implementation process responded to questions when other professionals did not respond or when responses were incorrect; however, the researcher refrained from interrupting online discussions.

Module 5 facilitated the patient to look back at the plans he made in conjunction with the health professionals in module 1 (Figure 2). Patients could examine the forms completed in module 1 and prepare for the next consultation with their health professional. The patient received these personalized messages underwritten with the name of their health care professional. Patients could see their progress in reducing the number of their cardiovascular risk factors, increasing physical activity levels, and implementing behavioral changes displayed in the same way as in module 1. The patient could read the physical activity recommendations provided by their health professional and practice them at home or elsewhere.

The health professionals could use all the modules of the PIB2 intervention with a log-in code. However, patients could only access module 5 with their log-in code. Health professionals could access background information on the PIB2 intervention and specific information for each module. Information on physical activity, physical fitness, general physical activity devices, making an activity plan, and cardiovascular risk factors were accessible. For patients, information about the PIB2 intervention with specific information on only module 5 was available. Background information and a response form for members of the general public visiting the website was also available.

### Intervention Mapping Step 5: Adoption and Implementation Plan

In step 5 of Intervention Mapping we developed a plan to facilitate implementation of the intervention (Table 7). Health professionals were invited to participate via a personalized email. They were invited to attend a meeting at which the Web-based intervention was demonstrated and could be practiced. The intervention was self-explanatory, and participants could choose not to attend the meeting and use the demonstration tool on the website to familiarize themselves with the use of the website. This was followed by telephone calls, emails, and meetings [50]. Much effort was put in motivating the health professionals to use and keep using the website. The health professionals selected the patients with cardiovascular risk factors. Health professionals were strongly recommended to use the intervention for every patient suitable for intervention.

**Table 7:** Intervention Mapping step 5 (adoption and implementation plan) and step 6 (evaluation plan).

Group		Design				
**Health professionals**						
	Intervention group 1	T1 Preintervention	T2 Start intervention, continuous measurement	T3 End intervention at 12 months		
	Control group A	T1 Preintervention I		T2 Preintervention II	T3 Start intervention, continuous measurement	T4 End of the intervention at 24 months
**Patients with cardiovascular risk factors**						
	Intervention group 2	T1 Preintervention	T2 Start intervention, continuous measurement	T3 End of the intervention at 12 months		
	Control group B			T1 Preintervention	T2 Start intervention, continuous measurement	T3 End of the intervention at 24 months

### Intervention Mapping Step 6: Evaluation Plan

The website was designed from the bottom up, beginning with determining the data needed to measure the effectiveness of the Web-based intervention [47]. The evaluation of the PIB2 intervention was designed according to the Consolidated Standards of Reporting Trials (CONSORT) criteria for reporting a randomized controlled trial [51] and will be reported in line with the emerging CONSORT-EHEALTH guideline [52]. The intervention will be evaluated by a pretest and posttest randomized controlled trial design consisting of a health professional intervention group and a health professional waiting-list control group (Table 7). The health professionals were randomly allocated to the intervention vs the waiting-list control group. The health professionals selected the patients with cardiovascular risk factors. Continuous data collection will be part of the Web-based intervention for a follow-up period of 12 months.

The effect evaluation will be performed to verify whether the PIB2 intervention was successful in extending the encouraging behavior of health professionals (if methods, conditions, and strategies to use these methods were successfully applied to change performance and change objectives). The effect evaluation will also be performed to verify whether the intervention was successful in strengthening the physical activity behavior of patients with cardiovascular risk factors (if we attained their performance and change objectives). For patients, the main outcome measure was improvement in cardiovascular risk profiles (if patients decreased their number of cardiovascular risk factors by at least one risk factor and/or decreased their levels of cardiovascular risk factors at the end of the intervention). The process evaluation will be performed during implementation of the PIB2 intervention through the collection of data on the use and usability of the PIB2-intervention modules.

## Discussion

This paper describes the systematic development process of the Web-based PIB2 intervention to disclose the black box of Web-based interventions and support future Web-based intervention development. Intervention Mapping step 1, the needs assessment for health professionals, indicated that we could state the problem that they do not always encourage patients with cardiovascular risk factors to become and/or stay physically active. The outcome measure for health care professionals was to extend their professional behavior. For patients at risk for cardiovascular disease, we could state the problem that they had low intentions toward, and inadequate levels of, physical activity and physical fitness. The outcome measure for patients with cardiovascular risk factors was to expand their risk-reduction behavior. Intervention Mapping step 2 resulted in matrices with specific performance and change objectives, for both health professionals and patients, linked with important social-cognitive determinants. Intervention mapping step 3 resulted in the linking of important social-cognitive determinants of intention and behavior, performance and change objectives with theory-based methods, and conditions and strategies to use these methods based on results of previous studies [39]. In step 4 of Intervention Mapping, we designed and pretested the website, and in step 5, the adoption and implementation plan of how to select and invite the health professionals and patients with cardiovascular risk factors to take part in the intervention was described. Step 6 Intervention Mapping resulted in the evaluation design for the intervention, consisting of a health professional intervention group and a health professional waiting-list control group. The intervention was implemented and the subgroups will be analyzed for their primary outcomes, extended professional behavior, and expanded patients’ risk-reduction behavior.

Changing intentions and behavior among both health professionals and patients is a complex process with many inhibiting factors. By assessing the needs of both health professionals and patients, we defined important and changeable social-cognitive determinants. Using evidence-based behavior change techniques in the development process of the intervention was important because they provided insight regarding how social-cognitive determinants of health professionals and patients’ may be changed. It proved difficult in Intervention Mapping step 3 (theory-based methods and strategies) to tune in on both groups, health professionals and patients with cardiovascular risk factors. Also in Intervention Mapping step 4, the development of the Web-based intervention made this complicated. We designed a prototype of the website, and it took many revisions, especially for the modules directed at health care professionals, before completion. The ease-of-use of the website for the selected methods proved complicated, as was the interaction between modules. Although influencing the professional behavior for health professionals and the physical activity behavior for patients with cardiovascular risk factors is difficult to achieve, the Intervention Mapping protocol provided us with tools to handle this complicated process.

Although it is easy to conclude that when you want to change the health-related behavior of patients it is a prerequisite that health professionals are able to handle the process of behavior change, this proved difficult. Choosing a Web-based intervention showed many opportunities to handle this complicated process, in our view more than any other method, but it proved difficult to make the website easy to use without having to explain everything.

Furthermore, although we carefully developed and tested the Web-based intervention in close cooperation with health professionals and patients with cardiovascular risk factors, much of its success depends on its feasibility and usefulness in the health professionals’ daily practice and on whether there is sufficient time and expertise to use the intervention during the consultation period.

## Abbreviations

CONSORT: Consolidated Standards of Reporting Trials
PIB2: Professional and Patient Intention and Behavior
SQUASH: short questionnaire to assess health-enhancing physical activity
